# An association between subcutaneous fat mass accumulation and hypertension

**DOI:** 10.1002/jgf2.427

**Published:** 2021-03-04

**Authors:** Kento Goto, Hirohide Yokokawa, Hiroshi Fukuda, Mizue Saita, Chieko Hamada, Teruhiko Hisaoka, Toshio Naito

**Affiliations:** ^1^ Department of General Medicine Juntendo University School of Medicine Tokyo Japan

**Keywords:** arteriosclerosis, hypertension, lifestyle‐related disorder, prevention, subcutaneous fat

## Abstract

Evidence to assess relationships between subcutaneous fat area (SFA) and lifestyle‐related diseases, including hypertension, remains limited. The aim of this study was to investigate the relationship between SFA and hypertension.

This study was a single‐institution, cross‐sectional study of 1,899 eligible Japanese participants who underwent health checkups between December 2016 and December 2018. All patients were measured for SFA and visceral fat area (VFA) by abdominal computed tomography (CT). SFA was divided into quartiles by gender, and multivariate logistic regression analysis was performed to estimate associations between SFA quartiles (Q) and hypertension.

Mean age and SFA were 60.9 9 (standard devastation [SD]:12.0) years and 123.0 (56.9) cm^2^ in men, and 60.6 (12.8) years and 146.6 (79.0) cm^2^ in women, respectively. Risk of hypertension from multivariate regression modeling compared with the lowest quartile (Q) in both sexes was as follows: for men Q2 [odds ratio (OR), 1; 95% confidence interval (CI), 0.55‐1.51 ], Q3 (OR, 1.73; 95%CI, 1.17‐2.56), and Q4 (OR, 1.96; 95%CI, 1.31‐2.94); for women Q2 (OR, 0.87; 95%CI, 0.48‐1.58), Q3 (OR, 1.73; 95%CI, 1.02‐2.95), and Q4 (OR, 2.54; 95%CI, 1.51‐4.28). The optimal SFA cutoff value at risk of hypertension was 114.7 cm^2^ in men and 169.3 cm^2^ in women.

The prevalence of hypertension was positively associated with SFA quartiles in both genders. The present results may indicate the necessity of considering not only VFA, but also SFA for the primary and secondary prevention of hypertension.

## Graphical abstract

This study examined the association between subcutaneous fat area (SFA) and hypertension. SFA was divided into quartiles by gender, and multivariate logistic regression analysis was performed to estimate associations between SFA quartiles and hypertension. There was a positive correlation between subcutaneous fat area quartile and hypertension.

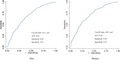

## 1 INTRODUCTION

Elevated blood pressure is the leading risk factor for cardiovascular and chronic kidney disease, and the estimated number of hypertensive individuals worldwide doubled between 1975 and 2015^1^. Excess salt intake was widely well known as one of the classical risk factors of increased blood pressure^2^, ^3^, ^4^. In recent years, the impact of obesity on elevated blood pressure has been received considerable attention^5^. Similarly, between 1975 and 2014, obesity became a public health problem worldwide^6^. In Japan, as in other high‐income countries, the number of presumed hypertension patients has been decreasing^7^. Nevertheless, an estimated 43 million Japanese have hypertension, and the treatment achievement rate is only 30.2%. Hypertension remains an important risk factor for cardiovascular diseases and poses a major public health challenge in Japan, as in other countries around the world^8^.

Obesity has been on the rise in Japan according to both 1956‐2005 and 1995‐2011 national surveys^9^, ^10^. Hypertension without obesity accounts for more than half of cases in Japan, but the increasing proportion of hypertension with obesity is also an issue, particularly among young‐ to middle‐aged men. Patients with this type of hypertension are considered prone to transition to metabolic syndrome (MetS). In recent years, MetS also has attracted attention as a risk factor for arteriosclerosis‐related cardiovascular disease, as well as hypertension^11^, ^12^. MetS is attributed to a combination of visceral fat‐type obesity, hypertension, hyperglycemia, and abnormal lipid metabolism. Different diagnostic criteria have been proposed, and ideas have varied from country to country. The pathogenic mechanisms are thought to be based on overlapping risk factors^13^, ^14^, leading from insulin resistance caused by visceral fat accumulation^11^, ^15^. In Japan, excess visceral fat accumulation is an essential item in the diagnostic criteria for Mets, and the standard criterion is a visceral fat area (VFA) ≥100 cm^2^. However, imaging tests such as CT and MRI are difficult to use for VFA measurement at all facilities because of problems of radiation exposure and examination costs, and waist circumference (WC) measurements (men ≥85 cm, women ≥90 cm) are therefore accepted for the diagnosis of central obesity^16^.

Subcutaneous fat area (SFA) accounts for the majority of body fat, but is not considered an independent risk factor for MetS. Various studies have examined the relationships between obesity and arteriosclerosis‐related cardiovascular factors among obese Caucasians^17^, ^18^, ^19^. However, studies involving suitably large cohorts of Asian individuals have been limited. Although several reports have suggested that SFA was independently associated with blood pressure, studies of wider age groups are needed^20^, ^21^. Although there have already been several reports on subcutaneous fat and dyslipidemia and glucose intolerance,^22^ the association between SFA and hypertension thus remains controversial.

This study aimed to investigate the association between SFA and hypertension, one of the components of MetS, by direct measurement from computed tomography (CT) to estimate abdominal adiposity.

## 2 MATERIALS AND METHODS

This cross‐sectional study surveyed 2,885 Japanese individuals who participated in a health checkup single institution in Tokyo, Japan, between December 2016 and December 2018. Participants with all of the physical measurements, blood tests, and abdominal CT were included in this study. Among these, 985 participants were excluded due to some missing data and one was excluded as a duplicate case. A final total of 1,899 participants were thus analyzed in the study as eligible cases.

Participants’ clinical data were retrospectively retrieved from single institutional database. All examinations included in this study were performed as part of the voluntary health checkup. The participants’ data were anonymized prior to the analysis. The Ethics Committee of the Juntendo University Hospital approved the study protocol (No. 18‐297), and written comprehensive informed consent was obtained consent from all participants when they were received health checkup.

Body weight, height, abdominal circumference, and body mass index (BMI) were measured with the participant in a standing position after changing into test clothes. BMI was calculated by dividing weight (kg) by height squared (m^2^). Systolic blood pressure (SBP) and diastolic blood pressure (DBP) were measured from the upper arm after the subject had been sitting at rest for ≥5 minutes. Serum and urine samples were collected from each subject after fasting overnight and were immediately submitted for biochemical analysis. A blood sample was used to determine total cholesterol (TC), high‐density lipoprotein‐cholesterol (HDL‐C), low‐density lipoprotein‐cholesterol (LDL‐C), triglycerides (TG), fasting plasma glucose (FPG), and glycosylated hemoglobin (HbA1c). LDL‐C levels were measured using the direct measurement method. HbA1c levels were determined by high‐performance liquid chromatography using an automated analyzer.

All participants underwent CT for measurement of SFA and VFA. These abdominal adipose distributions were measured on CT using a 320‐row CT system (Aquilion ONE / GENESIS Edition; Canon Medical), and "CT Fat Measurement" (Canon Medical) was used as body fat measurement software. Abdominal and subcutaneous fat areas were measured in the supine position at the level of the umbilicus during the expiratory delay phase according to the Japanese Obesity Practice Guidelines^23^. Fat surrounded by the inner surface of the abdominal wall was defined as VFA, and fat surrounded by the outer surface was defined as SFA. All tests were performed by staff trained at a single medical institution.

As part of the voluntary routine health checkup, participants were asked to complete self‐administered questionnaires regarding medical history (hypertension, dyslipidemia, diabetes), past other medical history, and health behaviors as listed in Breslow's seven health habits^24^. Breslow's seven health habits were non‐daily alcohol consumption, non‐smoker status, exercise at least ≥2 times/week for at least 30 minutes, BMI 18.5‐24.9 kg/m^2^, adequate sleep duration, daily breakfast consumption, and no snacking between meals^25^.

### 2.1 Definition of lifestyle‐related disorders

Lifestyle‐related disorders were defined as follows in this study. Hypertension was defined as SBP ≥140 mmHg, DBP ≥90 mmHg, or use of antihypertensive medications. Dyslipidemia was defined as TG ≥150 mg/dL, LDL‐C ≥140 mg/dL, HDL‐C <40 mg/dL, or use of anti‐dyslipidemia medications. Diabetes mellitus was defined as FGP ≥126 mg/dL or HbA1c ≥6.5%, or use of antidiabetic medications based on American Diabetes Association (ADA) diagnostic criteria^26^.

### 2.2 Statistical analysis

Results were analyzed by gender. SFA was stratified into quartiles by gender: men, Q1 (≤84.9 cm), Q2 (85.0‐114.9 cm), Q3 (115.0‐154.9 cm), and Q4 (≥155.0 cm); and women, Q1 (≤89.9 cm), Q2 (90.0‐134.9 cm), Q3 (135.0‐189.9 cm), and Q4 (≥190.0 cm). The two‐tailed Jonckheere‐Terpstra test for continuous variables and Cochran‐Armitage test for categorical variables were used to assess trends in *P*‐values across quartiles of SFA. Receiver operating characteristic curve analysis was used to assess appropriate cutoff values of SFA, and we estimated areas under the curve (AUCs) and measured the sensitivity and specificity of hypertension by SFA in both genders.

Multivariate logistic regression analysis was used to estimate associations between the presence of hypertension and SFA quartiles adjusted for age, lipid‐related factors, and snacking habits. All analyses were performed using Stata version 16 software (StataCorp LLC, College Station). Continuous variables are provided as mean (standard deviation). Values on both sides of *P* < 0.05 were considered statistically significant. The manuscript was written based on STROBE checklist.

## 3 RESULTS

The baseline characteristics of eligible participants in this study are shown in Table 1. Mean age was 60.9 (standard deviation [SD]:12.0) years for men and 60.6 (12.8) years for women. Mean SFA was 123.0 (56.9) cm^2^ for men and 146.6 (79.0) cm^2^ for women. Frequency of hypertension was 29.6% in men and 19.5% in women. Frequencies of dyslipidemia and diabetes mellitus were 43.8% and 17.2% in men, and 35.4% and 6.4% in women, respectively.

**TABLE 1:** Baseline participant characteristics

	Number (%) or mean (standard deviation)					
	Men (n = 1046)			Women (n = 853)		
	Non‐HT (n = 736)	HT (n = 310)	*P^a^*	Non‐HT (n = 687)	HT (n = 166)	*P* ^a^
Age (years)	59.6 (12.5)	64.0 (10.2)	<0.01	59.2 (12.9)	66.4 (10.4)	<0.01
Anthropometric measurements						
Body mass index (BMI) (kg/m^2^)	24.2 (3.1)	25.1 (3.4)	<0.01	21.6 (3.4)	23.4 (3.7)	<0.01
Waist circumference (WC) (cm)	86.4 (8.3)	89.1 (9.2)	<0.01	80.7 (9.7)	84.7 (10.0)	<0.01
Visceral fat area (cm^2^)	93.1 (47.1)	108 (47.2)	<0.01	58.6 (37.6)	81.0 (41.8)	<0.01
Visceral fat area ≧100(cm^2^)	305 (41.4)	168 (54.2)	<0.01	95 (13.8)	41 (24.7)	<0.01
Subcutaneous fat area (cm^2^)	119.5 (54.8)	131.2 (61.0)	<0.01	141.2 (78.0)	169.0 (79.3)	<0.01
Blood pressure‐related factors						
Systolic blood pressure (mmHg)	118.0 (11.0)	135 (14.2)	<0.01	113.4 (13.0)	134.8 (13.4)	<0.01
Diastolic blood pressure (mmHg)	72.0 (9.0)	81.2 (11.4)	<0.01	68.2 (9.0)	80.9 (10.3)	<0.01
Lipid‐related items						
High‐density lipoprotein‐cholesterol (HDL‐C) (mg/dL)	55.6 (14.5)	55.1 (13.8)	0.70	68.4 (15.5)	65.8 (15.5)	0.04
Low‐density lipoprotein‐cholesterol (LDL‐C) (mg/dL)	114.4 (29.5)	110.0 (26.6)	0.02	118.4 (28.8)	117.7 (28.4)	0.96
Triglycerides (TG) (mg/dL)	126.6 (86.0)	128.4 (81.2)	0.16	88.0 (48.1)	109.4 (67.1)	<0.01
Dyslipidemia	314 (42.7)	92 (29.7)	0.29	228 (33.2)	74 (44.6)	<0.01
Glucose‐related item						
Hemoglobin A1c (HbA1c) (%)	5.9 (0.7)	6.0 (0.6)	<0.01	5.7 (0.4)	5.9 (0.5)	<0.01
Diabetes mellitus	103 (14.0)	47 (15.2)	<0.01	35 (5.1)	20 (12.0)	<0.01
Healthy lifestyle characteristics						
Alcohol consumption (non‐daily drinker)	593 (80.6)	116 (37.4)	<0.01	627 (91.3)	145 (87.3)	0.16
Smoking behavior (non‐current smoker)	296 (40.2)	108 (34.8)	<0.01	375 (54.6)	128 (77.1)	<0.01
Exercise frequency (≧2 times per week)	107 (14.5)	35 (11.3)	<0.01	103 (15.0)	41 (24.7)	<0.01
Body mass index (18.5‐24.9 kg/m^2^)	456 (62.0)	56 (18.1)	<0.01	481 (70.0)	107 (64.5)	0.20
Hours of sleep (6‐9 h)	297 (49.4)	98 (31.6)	<0.01	310 (45.1)	102 (61.4)	<0.01
Breakfast (every morning)	301 (49.0)	105 (33.9)	<0.01	319 (46.4)	117 (70.5)	<0.01
Snacking between meals (none)	247 (33.6)	85 (27.4)	<0.01	265 (38.6)	89 (53.6)	<0.01
Total number of healthy lifestyle items	3.1 (1.8)	3.9 (1.7)	<0.01	3.6 (1.8)	4.4 (1.6)	<0.01

Note

Values are presented as mean ± standard deviation or number (%).

^a^ Student's *t* test for continuous variables and the chi‐squared test were used for categorical variables for comparisons between groups.

Table 2 shows the SFA quartile‐stratified characteristic SFA quartile in men, and Table 3 shows those in women. BMI, WC, VFA, blood pressure‐related variables, and lipid‐related variables all correlated positively with SFA in both sexes. A positive correlation was seen between SFA and total BMI and total score of healthy lifestyle characteristics in men, and between hypertension and diabetes‐related variables and alcohol intake in women.

**TABLE 2:** Sub Fat Area: Specific Characteristics with Man

	Number (%) or mean (standard deviation)				
Subcutaneous fat area (cm^2^)	Q1 ≤ 84.9	85.0 ≦ Q2 ≤ 114.9	115.0 ≦ Q3 ≤ 154.9	155.0 ≦ Q4	*P* ^a^
	(N = 280)	(N = 234)	(N = 270)	(N = 262)	
Age (years)	63.6 ± 12.1	61.8 ± 11.6	61.1 ± 11.3	57.1 ± 12.0	<0.01
Anthropometric measurements					
Body mass index (BMI) (kg/m^2^)	21.6 ± 2.1	23.6 ± 1.8	25.0 ± 2.0	27.7 ± 3.1	<0.01
Waist circumference (WC) (cm)	79.0 ± 5.9	84.7 ± 4.5	89.1 ± 5.2	96.1 ± 7.6	<0.01
Visceral fat area (cm^2^)	61.9 ± 37.2	96.6 ± 37.2	107.1 ± 41.1	126.6 ± 47.8	<0.01
Blood pressure‐related factors					
Systolic blood pressure (mmHg)	120.0 ± 14.6	122.9 ± 14.6	124.8 ± 14.3	124.8 ± 13.2	<0.01
Diastolic blood pressure (mmHg)	71.9 ± 10.6	74.4 ± 10.4	75.3 ± 10.4	77.5 ± 10.5	<0.01
Hypertension (present) (%)	74 (26.4)	58 (24.8)	90 (33.3)	88 (33.6)	0.02
Lipid‐related items					
High‐density lipoprotein‐cholesterol (HDL‐C) (mg/dL)	61.2 ± 15.7	54.6 ± 13.3	55.0 ± 13.4	50.6 ± 12.3	<0.01
Low‐density lipoprotein‐cholesterol (LDL‐C) (mg/dL)	107.1 ± 28.1	110.4 ± 26.7	117.3 ± 27.6	117.5 ± 30.9	<0.01
Triglycerides (TG) (mg/dL)	106.5 ± 82.7	127.9 ± 77.6	128.5 ± 81.2	147.1 ± 90.6	<0.01
Dyslipidemia (Present) (%)	114 (40.7)	110 (47.0)	126 (46.7)	144 (55.0)	<0.01
Glucose‐related items					
Hemoglobin A1c (HbA1c) (%)	5.9 ± 0.7	5.9 ± 0.6	5.9 ± 0.6	6.0 ± 0.7	0.26
Diabetes mellitus	56 (20.0)	37 (15.8)	42 (15.6)	51 (19.5)	0.69
Healthy lifestyle characteristics					
Alcohol consumption (non‐daily drinker)	135 (48.2)	176 (75.2)	209 (77.4)	204 (77.9)	0.52
Smoking behavior (non‐current smoker)	148 (52.9)	119 (50.9)	116 (43.0)	126 (48.1)	0.1
Exercise frequency (≧2 times per week)	55 (19.6)	37 (15.8)	48 (17.8)	41 (15.6)	0.32
Body mass index (18.5‐24.9 kg/m^2^)	140 (50.0)	182 (77.8)	141 (52.2)	42 (16.0)	<0.01
Hours of sleep (6‐9 h)	137 (48.9)	112 (47.9)	116 (43.0)	114 (43.5)	0.12
Breakfast (every morning)	143 (51.1)	115 (49.1)	126 (46.7)	122 (46.6)	0.24
Snacking between meals (none)	128 (45.7)	96 (41.0)	98 (36.3)	99 (37.8)	0.03
Total number of healthy lifestyle items	3.8 ± 1.8	3.6 ± 1.8	3.2 ± 1.7	2.9 ± 1.7	<0.01

^a^ *P*‐values for trend were estimated using the Jonckheere‐Terpstra test for continuous items and the Cochran‐Armitage two‐sided test for categorical items.

**TABLE 3:** Sub fat Area: Specific Characteristics with Woman

	Number (%) or mean (standard deviation)				
Subcutaneous fat area (cm^2^)	Q1 ≤ 89.9	90.0 ≦ Q2 ≤ 134.9	135.0 ≦ Q3 ≤ 189.9	190.0≦Q4	*P* ^a^
	(N = 218)	(N = 194)	(N = 220)	(N = 221)	
Age (years)	59.2 ± 13.1	60.2 ± 13.4	62.5 ± 12.2	60.3 ± 12.1	0.22
Anthropometric measurements					
Body mass index (BMI) (kg/m^2^)	18.8 ± 2.0	20.9 ± 2.2	22.4 ± 2.0	25.6 ± 3.4	<0.01
Waist circumference (WC) (cm)	71.8 ± 5.4	78.2 ± 6.0	83.2 ± 5.9	92.0 ± 8.1	<0.01
Visceral fat area (cm^2^)	30.3 ± 22.6	55.4 ± 33.5	70.5 ± 29.0	94.5 ± 39.0	<0.01
Blood pressure‐related factors					
Systolic blood pressure (mmHg)	114.3 ± 16.1	115.3 ± 14.7	119.6 ± 15.6	120.9 ± 14.7	<0.01
Diastolic blood pressure (mmHg)	69.4 ± 11.2	69.5 ± 9.7	71.2 ± 10.5	72.7 ± 10.2	<0.01
Hypertension (present) (%)	29 (13.3)	26 (13.4)	51 (23.2)	60 (27.1)	<0.01
Lipid‐related items					
High‐density lipoprotein‐cholesterol (HDL‐C) (mg/dL)	74.6 ± 15.4	69.6 ± 16.8	66.2 ± 14.0	61.4 ± 12.7	<0.01
Low‐density lipoprotein‐cholesterol (LDL‐C) (mg/dL)	109.2 ± 26.5	117.6 ± 26.3	122.4 ± 30.9	123.8 ± 28.4	<0.01
Triglycerides (TG) (mg/dL)	74.5 ± 55.1	83.7 ± 36.0	101.0 ± 49.5	108.2 ± 59.9	<0.01
Dyslipidemia (Present) (%)	40 (18.3)	64 (33.0)	96 (43.6)	102 (46.2)	<0.01
Glucose‐related items					
Hemoglobin A1c (HbA1c) (%)	5.7 ± 0.4	5.7 ± 0.4	5.8 ± 0.5	5.8 ± 0.5	<0.01
Diabetes mellitus	6 (2.8)	8 (4.1)	16 (7.3)	25 (11.3)	<0.01
Healthy lifestyle characteristics					
Alcohol consumption (non‐daily drinker)	193 (88.5)	172 (88.7)	199 (90.5)	208 (94.1)	0.04
Smoking behavior (non‐current smoker)	124 (56.9)	113 (58.2)	132 (60.0)	135 (61.1)	0.29
Exercise frequency (twice a week or more)	35 (16.1)	38 (19.6)	38 (17.3)	34 (15.4)	0.63
Body mass index (18.5‐24.9 kg/m^2^)	117 (53.7)	162 (83.5)	197 (89.5)	112 (50.7)	0.89
Hours of sleep (6‐9 h)	106 (48.6)	91 (46.9)	106 (48.2)	109 (49.3)	0.83
Breakfast (every morning)	113 (51.8)	104 (53.6)	111 (50.5)	108 (48.9)	0.43
Snacking between meals (none)	92 (42.2)	99 (51.0)	85 (38.6)	78 (35.3)	0.03
Total number of healthy lifestyle items	3.6 ± 1.8	4.0 ± 1.8	3.9 ± 1.8	3.5 ± 1.7	0.77

^a^ *P*‐values for trend were estimated using the Jonckheere‐Terpstra test for continuous items and the Cochran‐Armitage two‐sided test for categorical items.

**FIGURE 1:**
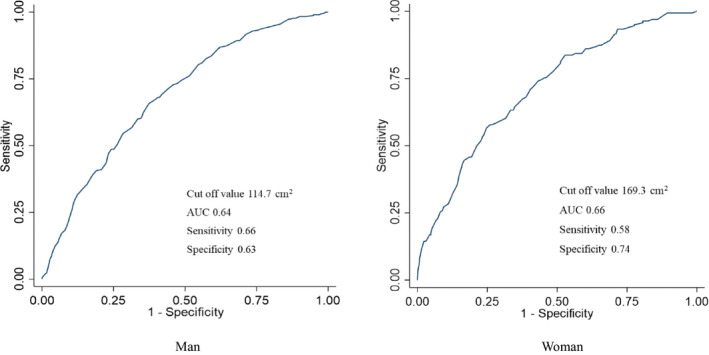
ROC curve analysis of subcutaneous fat area for hypertension

Table 4 shows the results of logistic regression analysis. Associations between hypertension and SFA quartiles were observed in both sexes after adjusting for related factors. The appropriate cutoff value, sensitivity, specificity, and AUC for SFA in men were 114.7 cm^2^, 0.66, 0.63, and 0.69, respectively. The appropriate cutoff value, sensitivity, specificity, and AUC for SFA in women were 169.3 cm^2^, 0.58, 0.74, and 0.71, respectively (Figure 1).

**TABLE 4:** Odds Ratios for Hypertension According to Subcutaneous Fat Mass (Logistic Regression Analysis)

	Bivariate^a^			Multivariate								
				Model 1^d^			Model 2^e^			Model 3^f^		
Subcutaneous fat area (cm^2^)	OR^b^	95% CI^c^	*P*	OR^b^	95% CI^c^	*P*	OR^b^	95% CI^c^	*P*	OR^b^	95% CI^c^	*P*
Elevated blood pressure: Man												
Q1 ≤ 84.9	Reference			Reference			Reference			Reference		
85.0 ≦ Q2 ≤ 114.9	0.97	0.65‐1.46	0.89	1.02	0.68‐1.55	0.92	0.94	0.62‐1.41	0.76	1	0.66‐1.51	0.99
115.0 ≦ Q3 ≤ 154.9	1.55	1.07‐2.26	0.02	1.78	1.21‐2.62	<0.01	1.51	1.03‐2.20	0.03	1.73	1.17‐2.56	<0.01
155.0 ≦ Q4	1.79	1.22‐2.65	<0.01	2.03	1.36‐3.02	<0.01	1.72	1.16‐2.54	<0.01	1.96	1.31‐2.94	<0.01
Elevated blood pressure: Woman												
Q1 ≤ 89.9	Reference			Reference			Reference			Reference		
90.0 ≦ Q2 ≤ 134.9	0.96	0.54‐1.72	0.9	0.91	0.50‐1.63	0.75	0.92	0.51‐1.66	0.79	0.87	0.48‐1.58	0.65
135.0 ≦ Q3 ≤ 189.9	1.75	1.05‐2.93	0.03	1.85	1.10‐3.12	0.02	1.64	0.97‐2.77	0.06	1.73	1.02‐2.95	0.04
190.0 ≦ Q4	2.48	1.50‐4.10	<0.01	2.71	1.62‐4.53	<0.01	2.33	1.39‐3.88	<0.01	2.54	1.51‐4.28	<0.01

^a^ Bivariate regression analysis was adjusted for age (10‐year increase).

^b^ odds ratio.

^c^ 95% confidence interval.

^d^ Model 1 was adjusted for age (10‐year increase) and no snacking habits.

^e^ Model 5 was adjusted for age (10‐year increase), current dyslipidemia.

^f^ Model 6 was adjusted for age (10‐year increase), current dyslipidemia, no snacking habits.

## 4 DISCUSSION

This cross‐sectional study showed the prevalence of hypertension was associated with increasing SFA in both genders, even after adjusting for associated factors. Appropriate cutoff values of SFA for the prevalence of HT in Japanese individuals might be 114.7 cm^2^ in men and 169.3 cm^2^ in women. To the best of our knowledge, little evidence has been gathered regarding the impact of subcutaneous adipose accumulation for hypertension among Japanese subjects.

The present results indicated a positive association between SFA and prevalence of hypertension. Several studies have stated that SFA seems to be associated with a lower risk of arteriosclerosis‐related diseases compared with VFA^27^, ^28^, ^29^. The frequency of gene expression for secretory proteins in adipose tissue is reportedly about 20% in subcutaneous fat and about 30% in visceral fat. The secretion of adipocytokines from subcutaneous fat influences visceral fat to a lesser extent. The reason is that obesity enhances insulin resistance, while increased leptin or adiponectin prevents fat accumulation. Visceral fat also makes more retinol‐binding protein than subcutaneous fat, reducing insulin sensitivity in the liver and other cells^22^. Therefore, the effect of subcutaneous fat‐type obesity seems relatively weak.

On the other hand, several studies have indicated the possibility of variability depending on ethnicity. The Ootori study evaluated visceral adipose tissue (VAT) and subcutaneous adipose tissue (SAT) in Japanese Americans, finding no significant association between subcutaneous fat and blood pressure^29^. Another study of Caucasian and African American women showed different results for African Americans^30^. Conversely, the Framingham Heart Study, primarily in Caucasians, and a large adult cohort study in China evaluated VAT and SAT, both of which correlated with metabolic risk, but VAT was more strongly associated than SAT^27^, ^31^. Our results were consistent with the findings from previous studies. In the present study, we examined the association between VFA and hypertension as well as SFA, and we found a significant association as previously reported (data not shown). And then, we conducted an additional multivariate analysis adjusting with both VFA and SFA. In the result, the significant association between SFA and hypertension was not observed because the association with VFA was stronger than SFA (data not shown). In spite of the results, excessive accumulation of SFA should still be considered to lead to an increased risk of hypertension, as visceral fat‐type obesity.

Some studies have calculated cutoff values for triceps and subscapular skinfold thickness^32^. In this study, an association between hypertension and subcutaneous fat thickness was found only in women. A report from Peru also acknowledged subscapular subcutaneous fat thickness as a risk factor for developing hypertension^33^. Similar to our study, subcutaneous fat in both sexes was significantly associated with risk of hypertension in that report. In these studies, subcutaneous fat thickness was measured by skinfold caliper instead of inspection of images from modalities such as dual‐energy X‐ray absorptiometry and CT for measuring fat thickness. That method of measurement is simpler than in our research, but the accuracy varies. Also, no studies appear to have calculated cutoff values for abdominal SFA. Whether this cutoff is effective needs to be verified in future analyses.

### 4.1 Limitations

This study has some limitations that should be considered. First, as a cross‐sectional observational study, causal relationships between SFA levels and hypertension could not be evaluated. Therefore, we will consider prospective cohort studies in the future. Second, the study was affected by selection bias. All participants were Japanese subjects who had undergone voluntary health checkups at a single institution in metropolitan area, and may have been inherently more aware of their health behaviors relative to residents in rural areas. Further multicenter analyses that include data from other populations are required. Third, some key data on items such as detailed information on medications, medication dosages, medication adherence, and status of menopause were not collected. Such data should be collected in future analyses. Finally, a self‐administered questionnaire was used to evaluate lifestyle habits, and some respondents may have under or overestimated their actual habits.

## 5 CONCLUSION

Our cross‐sectional study revealed significant associations between prevalence of hypertension and SFA quartiles after adjusting for confounders among participants undergoing voluntary health checkups. Better management of subcutaneous fat accumulation as well as visceral fat accumulation may be necessary for primary and secondary prevention of hypertension.

## CONFLICT OF INTEREST

The authors have stated explicitly that there are no conflicts of interest in connection with this article.

## ETHICAL STATEMENT

The Ethics Committee of Juntendo University reviewed and approved the research protocol using the retrospective data (No 18‐296), and written comprehensive informed consent was obtained from all participants when they were received health checkup.
